# Evaluation of the cardiovascular system in patients with juvenile dermatomyositis

**DOI:** 10.1186/1546-0096-9-S1-P54

**Published:** 2011-09-14

**Authors:** Violetta Opoka – Winiarska, Renata Jawniak, Andrzej Emeryk

**Affiliations:** 1 Dept. of Pediatric Pulmonology and Rheumatology, Medical University of Lublin, Poland; 2 Dept. of Pediatric Cardiology, Medical University, Lublin, Poland

## Background

Juvenile Dermatomyositis (JDM) is a systemic, autoimmune disease in which the main process underlying the pathogenesis is vasculitis. Recent reports suggest that subclinical heart disease is related to systemic nature of JIA.

## Aim

The aim of this study was the assessment of cardiac function in patients with JDM on the basis of an echocardiography, ECG and 24-hour ECG Holter Monitoring.

## Methods

8 patients at average age 11,12 (range 7 – 18) years, 3 girls and 5 boys with diagnosed JDM, all during treatment were included in the study. An average duration of illness at the time of study was 3,25 ± 1,83 years.

Disease activity and circulatory system were assessed by clinical examination. ECG, echocardiography and 24 hour ECG Holter Monitoring were performed and analysed. Disease activity was evaluated on a scale DAS for JDM.

## Results

Physical examination of patients did not show any cardiac symptoms. BP systolic and diastolic were normal. An average total Disease Activity Score evaluated on a scale DAS for JDM was 4,12 ± 2,94 (DAS for muscle was 0,75 ±1,03 and DAS for skin was 3,375 ± 2,44).

In all children,in examine based on echocardiography was found a small mitral valve insufficiency (from trace to the first degree). In one patient a mild pulmonary valve stenosis was diagnosed, in one - dilated cardiomyopathy, and in one patient foramen ovale. In a routine ECG and during the 24-hour Holter monitoring, accelerated atrio-ventricular conduction was recorded in 3 children. One patient was found with mild ventricular arrhythmia (200 single ventricular ectopic beats during the 24-hour monitoring period was recorded). In another patient was borderline QT interval (QTc = 0.45 s).

## Conclusion

In all patients varying degrees of cardiac abnormalities were detected. Because of frequent subclinical symptoms patients with JDM require cardiology consultation, even if they have no clinical signs of circulatory disorders.

